# Inference from longitudinal laboratory tests characterizes temporal evolution of COVID-19-associated coagulopathy (CAC)

**DOI:** 10.7554/eLife.59209

**Published:** 2020-08-17

**Authors:** Colin Pawlowski, Tyler Wagner, Arjun Puranik, Karthik Murugadoss, Liam Loscalzo, AJ Venkatakrishnan, Rajiv K Pruthi, Damon E Houghton, John C O'Horo, William G Morice, Amy W Williams, Gregory J Gores, John Halamka, Andrew D Badley, Elliot S Barnathan, Hideo Makimura, Najat Khan, Venky Soundararajan

**Affiliations:** 1 nference, incCambridgeUnited States; 2 Mayo ClinicRochesterUnited States; 3 Mayo Clinic LaboratoriesRochesterUnited States; 4 Mayo Clinic PlatformRochesterUnited States; 5 Janssen pharmaceutical companies of Johnson & Johnson (J&J)Spring HouseUnited States; Radboud University Medical CenterNetherlands; Radboud University Medical CenterNetherlands

**Keywords:** laboratory tests, COVID-19, electronic health record (EHR), SARS-CoV-2, coagulation, thrombolytic agents, Human

## Abstract

Temporal inference from laboratory testing results and triangulation with clinical outcomes extracted from unstructured electronic health record (EHR) provider notes is integral to advancing precision medicine. Here, we studied 246 SARS-CoV-2 PCR-positive (COVID_pos_) patients and propensity-matched 2460 SARS-CoV-2 PCR-negative (COVID_neg_) patients subjected to around 700,000 lab tests cumulatively across 194 assays. Compared to COVID_neg_ patients at the time of diagnostic testing, COVID_pos_ patients tended to have higher plasma fibrinogen levels and lower platelet counts. However, as the infection evolves, COVID_pos_ patients distinctively show declining fibrinogen, increasing platelet counts, and lower white blood cell counts. Augmented curation of EHRs suggests that only a minority of COVID_pos_ patients develop thromboembolism, and rarely, disseminated intravascular coagulopathy (DIC), with patients generally not displaying platelet reductions typical of consumptive coagulopathies. These temporal trends provide fine-grained resolution into COVID-19 associated coagulopathy (CAC) and set the stage for personalizing thromboprophylaxis.

## Introduction

There is a growing body of evidence suggesting that severe COVID-19 outcomes may be associated with dysregulated coagulation (Tang et al., 2020), including stroke, pulmonary embolism, myocardial infarction, and other venous or arterial thromboembolic complications (Klok et al., 2020). This so-called COVID-19 associated coagulopathy (CAC) shares similarities with disseminated intravascular coagulation (DIC) and thrombotic microangiopathy but also has distinctive features (Levi et al., 2020). Given the significance of CAC to COVID-19 mortality, there is an urgent need for fine-grained resolution into the temporal manifestation of CAC, particularly in comparison to the broad-spectrum of other, better characterized coagulopathies. While there are studies suggesting associations between COVID-19 infection and mortality with thrombocytopenia, D-dimer levels, and prolongation of prothrombin time, the signatures of CAC onset and progression as well as their connection to clinical outcomes are not well defined (Tang et al., 2020; Gao et al., 2020; Panigada et al., 2020). An advanced understanding of this phenotype may aid in the risk stratification of patients, thus facilitating optimal monitoring strategies during disease evolution through the paradigm of precision medicine.

To this end, we instituted a holistic data science platform across an academic medical center that enables machine intelligence to augment the curation of phenotypes and outcomes from over 10 million electronic health record (EHR) clinical notes and associated 3.2 million lab tests from 2232 SARS-CoV-2 positive (COVID_pos_) and 72,354 confirmed SARS-CoV-2 negative (COVID_neg_) patients over a retrospectively defined 2-month observation period straddling the date of the PCR test. For the COVID_pos_ cohort, we center the 2-month observation period around the date of the first positive PCR test for SARS-CoV-2, and for the COVID_neg_ cohort, we center the 2-month observation period around the date of the first PCR test for SARS-CoV-2 (see Materials and methods). It is important to note that not all individuals infected by SARS-CoV-2 develop symptoms of COVID-19, but rather that a majority of patients are either asymptomatic or have mild-to-moderate symptoms not requiring hospitalization for COVID-19 (Wagner et al., 2020). Furthermore, the guidelines followed for PCR-testing included a routine screening of individuals, patients displaying COVID-19 symptoms as per the Mayo Clinic (Coronavirus disease, 2019) and CDC definitions (Website, 2020), and possibly contact with infected persons or underlying predisposing conditions (Wagner et al., 2020).

By compiling all available laboratory testing data for the 30 days preceding the first SARS-CoV-2 PCR positive diagnostic testing date (day 0), as well as the 30 days following the diagnostic testing date, and triangulating this information with medications and clinical outcomes, we were able to identify laboratory abnormalities significantly associated with the COVID_pos_ group. We identified coagulation-related parameters among this set of abnormalities and then studied aggregate as well as individual patient trajectories that could aid in extracting a temporal signature of CAC onset and progression. We also correlated these signals with the clinical outcomes of these patients.

In order to hone into longitudinal lab test trends that would apply at the individual patient level, we restricted our analysis to patients with available serial testing data, which had at least three test results of the same type during the observation period. After applying these inclusion criteria, 246 COVID_pos_ and 13,666 COVID_neg_ patients met study the inclusion criteria. The need for longitudinal data on the testing results, while constraining the study population size greatly, enables us to provide a fine-grained temporal resolution of CAC for the first time.

After filtering the patients with the available longitudinal testing data, the median age in the COVID_pos_ and COVID_neg_ groups were 60.8 years and 64.1 years, respectively (see Materials and methods and Table 1), and the numbers of males were 137 (56%) and 7129 (52%), respectively. The total numbers of pre-existing coagulopathies in the COVID_pos_ and COVID_neg_ groups were 31 (13%) and 3901 (29%), respectively. These counts of coagulopathies include the following phenotypes identified in the clinical notes from day −365 to day −31 relative to the PCR testing date: deep vein thrombosis, pulmonary embolism, myocardial infarction, venous thromboembolism, thrombotic stroke, cerebral venous thrombosis, and disseminated intravascular coagulation (see Table 1 for detailed breakdown). The number of COVID_pos_ patients hospitalized in the month prior to the SARS-CoV-2 PCR testing date was 41 (17%), compared to 1247 (9.1%) for the COVID_neg_ cohort.

**Table 1.** Summary of patient characteristics for the overall COVID_pos_, COVID_neg_ (matched), and COVID_neg_ cohorts. The COVID_neg_ (matched) cohort was constructed using 1:10 propensity score matching to balance each of the clinical covariates, including demographics (age, gender, race), medication use (anticoagulant/antiplatelet use in the preceding 30 days/1 year of PCR testing date), medical history of thrombotic events from the past year, and hospitalization status in the month prior to the date of PCR testing.

Patient characteristics	COVID_pos_	COVID_neg_ (matched)	COVID_neg_
Number of patients	246	2460	13,666
Age in years	60.8	60.9	64.1
Gender:			
Male	137 (56%)	1388 (56%)	7129 (52%)
Race:			
White	154 (63%)	1540 (63%)	12,241 (90%)
Black	24 (9.8%)	313 (13%)	569 (4.2%)
Asian	18 (7.3%)	207 (8.4%)	274 (2.0%)
American Indian	23 (9.3%)	81 (3.3%)	81 (0.59%)
Other	27 (11%)	319 (13%)	501 (3.7%)
Medication use in the preceding 30 days of PCR testing date:			
Anticoagulants	63 (26%)	596 (24%)	5171 (38%)
Antiplatelets	30 (12%)	298 (12%)	2230 (16%)
Medication use in the preceding 1 year of PCR testing date:			
Anticoagulants	86 (35%)	819 (33%)	7476 (55%)
Antiplatelets	40 (16%)	419 (17%)	3620 (26%)
Medical history of thrombotic events in 1 year prior to study period:			
Deep vein thrombosis	15 (6.1%)	153 (6.2%)	2,110 (15%)
Pulmonary embolism	12 (4.9%)	112 (4.6%)	1258 (9.2%)
Myocardial infarction	11 (4.5%)	142 (5.8%)	1468 (11%)
Venous thromboembolism	4 (1.6%)	44 (1.8%)	615 (4.5%)
Thrombotic stroke	1 (0.41%)	3 (0.12%)	143 (1.0%)
Cerebral venous thrombosis	0	1 (0.04%)	7 (0.05%)
Disseminated intravascular coagulation	0	1 (0.04%)	30 (0.22%)
Any thrombotic event	31 (13%)	308 (13%)	3901 (29%)
Hospitalized in the month prior to PCR testing date	41 (17%)	304 (12%)	1247 (9%)

To balance these clinical covariates and others between the two cohorts, we applied 1:10 propensity score matching to define a subset of 2460 patients from the COVID_neg_ cohort to use for the final statistical analysis (see Materials and methods). In particular, the general categories of covariates considered for balancing included: demographics, anticoagulant/antiplatelet medication use, medical history of pre-existing coagulopathies, and hospital admission status. Population-level characteristics of the COVID_pos_, COVID_neg_, and the final propensity score-matched COVID_neg_ (matched) cohorts are summarized in Table 1. We observe that the COVID_pos_ and COVID_neg_ (matched) cohorts are well-balanced along these covariates which are potential confounding variables for thrombotic events and coagulopathy-related lab tests during the study period.

## Results

### Longitudinal analysis identifies lab test results characteristic of COVID-19 at specific prognostic time intervals

To identify laboratory test results that differ between COVID_pos_ and COVID_neg_ (matched) patients, we analyzed longitudinal trends of 194 laboratory test results in the 30 days before and after the day of PCR testing (designated as day 0). As most patients did not undergo laboratory testing for each assay on a daily basis, we grouped the measurements into nine time windows reflecting potential stages of infection as follows: pre-infection (days −30 to −11), pre-PCR (days −10 to −2), time of clinical presentation (days −1 to 0), and post-PCR phases 1 (days 1 to 3), 2 (days 4 to 6), 3 (days 7 to 9), 4 (days 10 to 12), 5 (days 13 to 15), and 6 (days 16 to 30). We only considered test-time window pairs in which at least three patients contributing to laboratory test results in both groups. During each time window, we then compared the distribution of results from COVID_pos_ versus COVID_neg_ (matched) patients, allowing us to identify any lab tests which were significantly altered in COVID_pos_ patients during any time of disease acquisition, onset, and/or progression.

**Figure 1.**
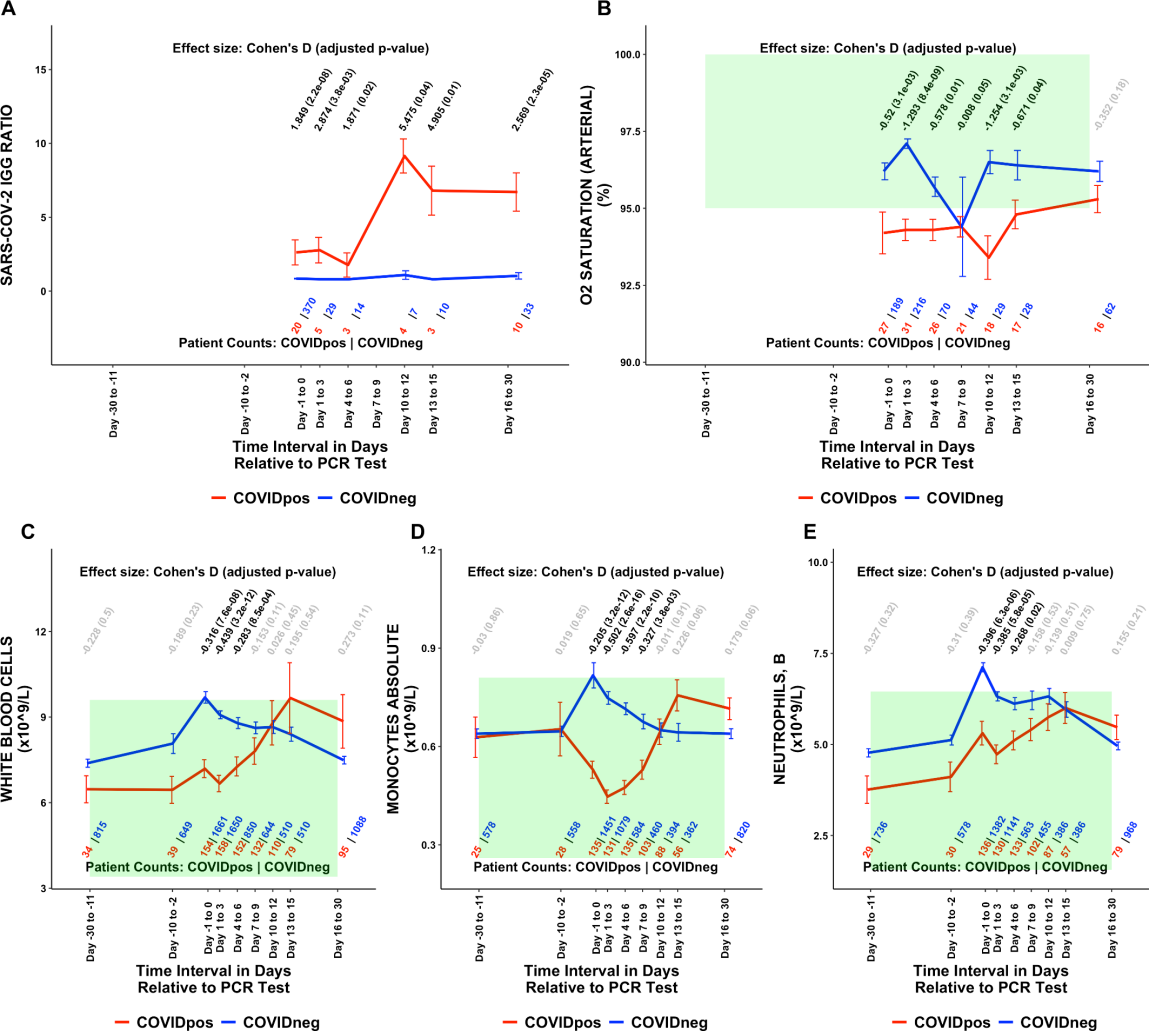
Longitudinal and temporally resolved analysis highlights positive control lab tests elevated in COVID_pos_ patients along with distinctive immune signatures. Longitudinal trends in COVID_pos_ versus COVID_neg_ (matched) patients for the following lab tests: (**A**) SARS-CoV-2 IGG ratio, (**B**) oxygen saturation in arterial blood, (**C**) white blood cells, (**D**) monocytes absolute, and (**E**) neutrophils, blood. For any window of time during which at least three patients in each cohort had test results, data are shown as mean with standard errors. The normal range for each lab test is shaded in green. Values given horizontally along the top of the plot are Cohen’s D statistics comparing the COVID_pos_ and COVID_neg_ (matched) cohorts along with the BH-adjusted Mann-Whitney test p-values. Significant differences (adjusted p-value <0.05) are shown in black, while non-significant values are shown in gray. Values given horizontally along the bottom of the plot are the numbers of patients in the COVID_pos_ and COVID_neg_ cohorts, respectively (i.e. # COVID_pos_ | # COVID_neg_). For certain lab tests, some data points are missing because these time windows had fewer than three data points in the COVID_pos_ cohort.

**Figure 2.**
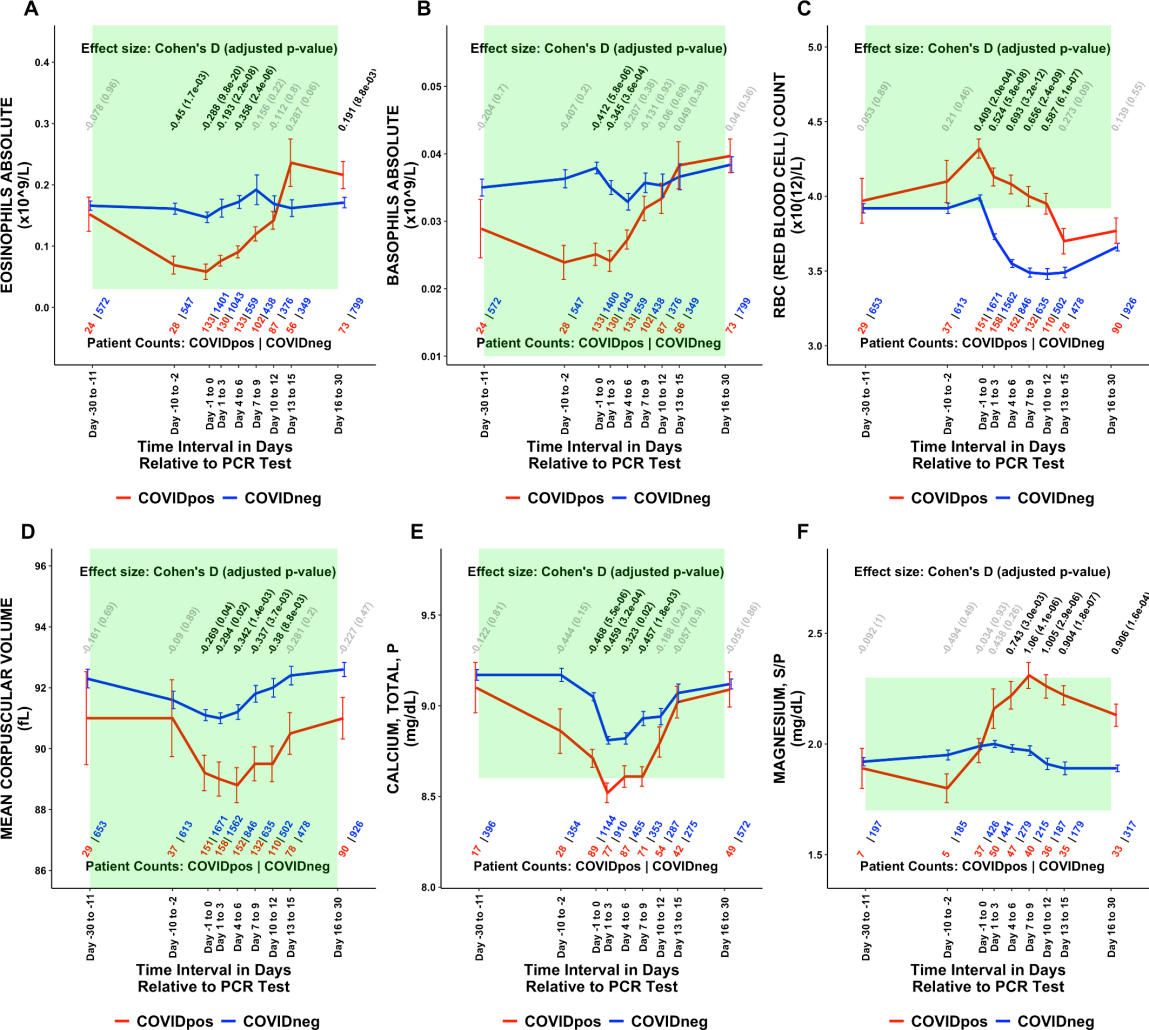
Longitudinal trends of COVID_pos_ patients’ lab tests show distinctive immune, hematologic, and serum chemistry signatures within normal ranges. Longitudinal trends in COVID_pos_ versus COVID_neg_ (matched) patients for the following lab tests: (**A**) eosinophils absolute, (**B**) basophils absolute, (**C**) red blood cell count, (**D**) mean corpuscular volume, (**E**) calcium total, plasma, and (**F**) magnesium total, serum/plasma. For any window of time during which at least three patients in each cohort had test results, data are shown as mean with standard errors. The normal range for each lab test is shaded in green. Values given horizontally along the top of the plot are Cohen’s D statistics comparing the COVID_pos_ and COVID_neg_ (matched) cohorts along with the BH-adjusted Mann-Whitney test p-values. Significant differences (adjusted p-value <0.05) are shown in black, while non-significant values are shown in gray. Values given horizontally along the bottom of the plot are the numbers of patients in the COVID_pos_ and COVID_neg_ cohorts, respectively (i.e. # COVID_pos_ | # COVID_neg_). For certain lab tests, some data points are missing because these time windows had fewer than three data points in the COVID_pos_ cohort.

**Figure 3.**
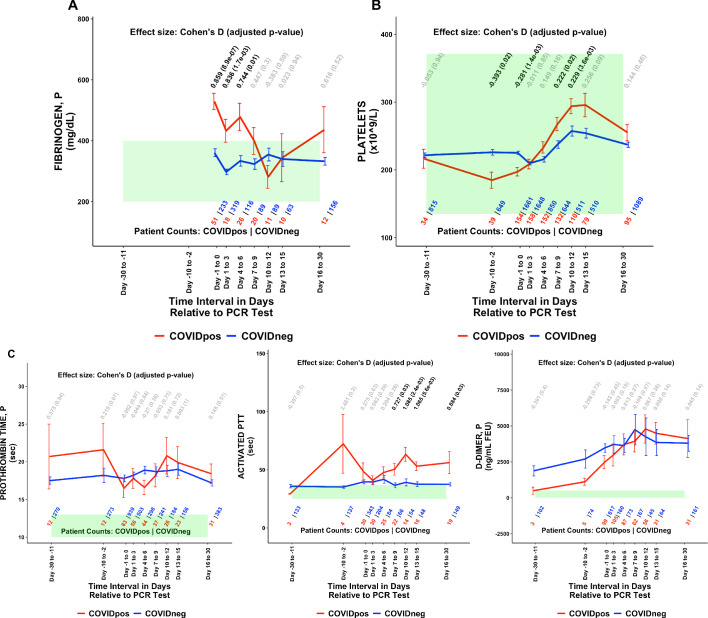
COVID_pos_ patients show distinctly opposite temporal trends in fibrinogen and platelet counts starting at the time of diagnosis. Longitudinal trends of COVID_pos_ versus COVID_neg_ (matched) patients for the following lab tests: (**A**) fibrinogen, plasma, (**B**) platelets, and (**C**) other coagulation-related tests including prothrombin time (PT), activated partial thromboplastic time (aPTT), and D-dimers. For any window of time during which at least three patients in each cohort had test results, data are shown as mean with standard errors. The normal range for each lab test is shaded in green. Values given horizontally along the top of the plot are Cohen’s D statistics comparing the COVID_pos_ and COVID_neg_ (matched) cohorts along with the BH-adjusted Mann-Whitney test p-values. Significant differences (adjusted p-value <0.05) are shown in black, while non-significant values are shown in gray. Values given horizontally along the bottom of the plot are the numbers of patients in the COVID_pos_ and COVID_neg_ cohorts, respectively (i.e. # COVID_pos_ | # COVID_neg_). For certain lab tests, some data points are missing because these time windows had fewer than three data points in the COVID_pos_ cohort.

Of the 1709 lab test-time window pairs with adequate data points for comparison, we identified 130 such pairs (comprising 66 unique lab tests) which met our thresholds for statistical significance (Cohen’s D >0.35, BH-adjusted Mann-Whitney p-value <0.05; Table 2). Among these were lab tests that may be considered positive controls for our analysis. From the time of clinical presentation onward, elevated titers of SARS-CoV-2 IgG antibodies (Figure 1A) and a reduction in blood oxygenation in COVID_pos_ patients were observed (Figure 1B). We also identified abnormalities in several other classes of lab tests, including immune cell counts (Figures 1C–E and 2A–B), red blood cell counts (Figure 2C), mean corpuscular volume (Figure 2D), calcium and magnesium levels (Figure 2E–F), and coagulation-related tests (Figure 3).

**Table 2.** Summary of lab tests significantly different between COVID_pos_ and propensity score-matched COVID_neg_ cohorts during at least one clinical time window. Data from individual patients were averaged over the defined time windows, and the mean values were compared between COVID_pos_ and COVID_neg_ patients. The lab test-time window pairs shown are those which met our defined thresholds for statistical significance and substantial effect (BH-adjusted Mann-Whitney p-value <0.05 and Cohen’s D absolute value >0.35). In particular, 130 of the initial 1709 (test, time window) pairs with at least one patient met these thresholds. Rows are sorted alphabetically by test and then time window (from earliest to latest). Coagulation-related tests of particular interest (fibrinogen, platelets, prothrombin time, activated partial thromboplastin time, and D-dimer) are highlighted in gray. Sample sources are denoted as: P = plasma, S = serum, S/P = serum/plasma, B = blood, U = urine.

Test	Units	Time window	Count COVID_pos_	Count COVID_neg_	Mean COVID_pos_	Mean COVID_neg_	Cohen's D	BH-adj M-W p-value
ABGRS pH Arterial	pH	Days 16–30 Post-Dx	18	91	7.45	7.4	0.775	0.02
ABGRS PO_2_ Arterial	mm Hg	Days 1–3 Post-Dx	16	204	81.9	129.6	−0.797	3.1E-03
ABGRS PO_2_ Arterial	mm Hg	Days 4–6 Post-Dx	25	82	78.1	113.2	−0.712	8.8E-03
ABGRS PO_2_ Arterial	mm Hg	Days 7–9 Post-Dx	23	58	77.2	121.9	−0.807	1.0E-03
ABGRS PO_2_ Arterial	mm Hg	Days 10–12 Post-Dx	18	37	76.4	104.2	−0.965	2.6E-03
ABGRS PO_2_ Arterial	mm Hg	Days 13–15 Post-Dx	15	31	73.1	112.3	−0.964	6.0E-03
Activated Partial Thrombopl Time, P	sec	Days 7–9 Post-Dx	22	66	50.5	36.7	0.727	0.026
Activated Partial Thrombopl Time, P	sec	Days 10–12 Post-Dx	14	54	63.3	39.2	1.085	2.4E-03
Activated Partial Thrombopl Time, P	sec	Days 13–15 Post-Dx	16	48	53.1	37.6	1.065	5.6E-03
Activated Partial Thrombopl Time, P	sec	Days 16–30 Post-Dx	19	149	56.2	37.5	0.884	0.027
Alanine Aminotransferase (ALT), P	U/L	Days 10–12 Post-Dx	27	104	77.3	46	0.512	0.015
Albumin, P	g/dL	Days 7–9 Post-Dx	42	188	3.06	3.41	−0.54	5.6E-03
Albumin, S/P	g/dL	Clinical presentation	85	812	3.43	3.81	−0.591	4.8E-06
Albumin, S/P	g/dL	Days 1–3 Post-Dx	77	525	3.26	3.6	−0.541	3.8E-05
Albumin, S/P	g/dL	Days 10–12 Post-Dx	61	254	3.35	3.66	−0.47	2.6E-03
Alkaline Phosphatase, P	U/L	Days 4–6 Post-Dx	42	139	88.8	126.7	−0.395	3.7E-03
Arterial O_2_ PP Diff	None	Clinical presentation	21	106	268.1	152.1	0.924	9.7E-03
Arterial O_2_ PP Diff	None	Days 1–3 Post-Dx	22	112	225.9	147.4	0.639	0.017
Arterial O_2_ PP Diff	None	Days 4–6 Post-Dx	17	49	271.4	155	0.891	4.8E-03
Aspartate Aminotransferase (AST), P	U/L	Days 10–12 Post-Dx	27	107	67.6	44.7	0.404	3.6E-04
Basophils Absolute	×10(9)/L	Clinical presentation	133	1400	0.0251	0.0379	−0.412	5.8E-06
Bicarbonate [MMOL/L] in Arterial Blood	mmol/L	Days 16–30 Post-Dx	18	91	28.6	24.3	0.857	7.6E-03
Bicarbonate in Arterial Blood	mmol/L	Days 1–3 Post-Dx	26	193	23.2	21.4	0.513	0.027
BUN, P	mg/dL	Days 16–30 Post-Dx	49	562	31.4	21.9	0.555	3.9E-03
C-reactive Protein Quantative, S	mg/L	Clinical presentation	85	666	100.2	68.2	0.375	6.8E-05
Calcium, Ionized, B	mg/dL	Clinical presentation	14	201	4.36	4.77	−0.67	0.015
Calcium, Ionized, B	mg/dL	Days 1–3 Post-Dx	18	270	4.42	4.73	−0.783	8.5E-04
Calcium, Total, P	mg/dL	Clinical presentation	89	1144	8.71	9.05	−0.468	5.5E-06
Calcium, Total, P	mg/dL	Days 1–3 Post-Dx	77	910	8.52	8.81	−0.459	3.2E-04
Calcium, Total, P	mg/dL	Days 7–9 Post-Dx	71	353	8.61	8.93	−0.457	1.8E-03
Calcium, Total, S	mg/dL	Clinical presentation	83	941	8.29	8.91	−0.854	1.9E-13
Calcium, Total, S	mg/dL	Days 1–3 Post-Dx	98	1025	8.28	8.77	−0.717	2.2E-10
Calcium, Total, S	mg/dL	Days 4–6 Post-Dx	87	568	8.4	8.69	−0.435	2.3E-03
Calcium, Total, S	mg/dL	Days 7–9 Post-Dx	82	433	8.49	8.76	−0.384	0.011
Carboxyhemoglobin, ARTERIAL	%	Clinical presentation	34	356	0.507	0.991	−0.71	2.0E-04
Carboxyhemoglobin, Arterial	%	Days 1–3 Post-Dx	44	436	0.535	0.9	−0.711	5.9E-05
Carboxyhemoglobin, Arterial	%	Days 4–6 Post-Dx	58	166	0.678	0.974	−0.544	3.0E-03
Carboxyhemoglobin, Arterial	%	Days 7–9 Post-Dx	45	102	0.704	0.97	−0.472	0.048
Carboxyhemoglobin, Venous	%	Days 1–3 Post-Dx	10	73	0.701	1.16	−0.862	0.02
Carboxyhemoglobin, Venous	%	Days 4–6 Post-Dx	14	47	0.725	1.29	−0.837	3.7E-03
Chloride, P	mmol/L	Days 1–3 Post-Dx	77	906	100.1	101.9	−0.363	7.7E-03
Eosinophils Absolute	×10(9)/L	Pre-diagnosis	28	547	0.0689	0.161	−0.45	1.7E-03
Esosinophils Absolute	×10(9)/L	Days 4–6 Post-Dx	133	559	0.0906	0.172	−0.358	2.4E-06
Fibrinogen, P	mg/dL	Clinical presentation	51	233	528.9	360.7	0.859	8.9E-07
Fibrinogen, P	mg/dL	Days 1–3 Post-Dx	18	319	432.6	297.4	0.836	1.7E-03
Fibrinogen, P	mg/dL	Days 4–6 Post-Dx	26	116	477.8	333.7	0.744	0.014
Glucose, Random, S	mg/dL	Days 13–15 Post-Dx	49	314	150	126.5	0.544	0.013
Hematocrit, B	%	Days 1–3 Post-Dx	158	1582	36.5	33.8	0.433	9.6E-06
Hematocrit, B	%	Days 4–6 Post-Dx	152	851	36	32.1	0.621	2.2E-10
Hematocrit, B	%	Days 7–9 Post-Dx	132	639	35.5	31.8	0.587	5.8E-08
Hematocrit, B	%	Days 10–12 Post-Dx	110	505	35.1	31.8	0.511	1.7E-05
Hemoglobin Arterial	g/dL	Days 1–3 Post-Dx	31	208	12.1	10.8	0.651	0.025
Hemoglobin, B	g/dL	Days 1–3 Post-Dx	158	1682	11.9	11.1	0.358	2.2E-04
Hemoglobin, B	g/dL	Days 4–6 Post-Dx	152	873	11.8	10.4	0.636	1.4E-10
Hemoglobin, B	g/dL	Days 7–9 Post-Dx	132	653	11.6	10.4	0.56	2.0E-07
Hemoglobin, B	g/dL	Days 10–12 Post-Dx	110	516	11.4	10.3	0.49	2.6E-05
Ionized Calcium, Arterial	mg/dL	Days 16–30 Post-Dx	8	36	4.93	4.48	1.561	0.022
Lactate Dehydrogenase, S	U/L	Days 10–12 Post-Dx	21	88	406.2	295.2	0.463	1.4E-03
Lactate, P	mmol/L	Clinical presentation	89	954	1.37	1.93	−0.462	3.1E-06
Lymphocytes Percent	%	Days 13–15 Post-Dx	5	66	33.2	15	1.514	0.048
Lymphs Absolute	×10(9)/L	Days 13–15 Post-Dx	56	349	3.12	1.11	0.44	0.018
Magnesium, Plasma	mg/dL	Days 10–12 Post-Dx	20	87	2.14	1.91	0.772	0.015
Magnesium, S/P	mg/dL	Days 4–6 Post-Dx	47	279	2.22	1.98	0.743	3.0E-03
Magnesium, S/P	mg/dL	Days 7–9 Post-Dx	40	215	2.31	1.97	1.06	4.1E-06
Magnesium, S/P	mg/dL	Days 10–12 Post-Dx	36	187	2.26	1.91	1.005	2.9E-06
Magnesium, S/P	mg/dL	Days 13–15 Post-Dx	35	179	2.22	1.89	0.904	1.8E-07
Magnesium, S/P	mg/dL	Days 16–30 Post-Dx	33	317	2.13	1.89	0.906	1.6E-04
Manual Diff Promyelocytes	%	Days 1–3 Post-Dx	6	55	0.25	0	1.402	0.027
Mean Corpuscular Volume	fL	Days 10–12 Post-Dx	110	502	89.5	92	−0.38	8.8E-03
Methemoglobin, ABG	%	Clinical presentation	34	356	0.335	0.571	−0.629	6.0E-03
Methemoglobin, ABG	%	Days 1–3 Post-Dx	44	436	0.425	0.697	−0.463	1.5E-03
Monocytes Absolute	×10(9)/L	Days 1–3 Post-Dx	131	1079	0.447	0.748	−0.502	2.6E-16
Monocytes Absolute	×10(9)/L	Days 4–6 Post-Dx	135	584	0.475	0.715	−0.597	2.2E-10
N-terminal-PRO-Brain Type Natriuretic Peptide, S	pg/mL	Days 4–6 Post-Dx	10	63	415.6	7609.7	−0.525	2.9E-03
Neutrophils, B	×10(9)/L	Clinical presentation	136	1382	5.31	7.12	−0.396	6.3E-06
Neutrophils, B	×10(9)/L	Days 1–3 Post-Dx	130	1141	4.73	6.32	−0.385	5.8E-05
NT-PRO BNP, P	pg/mL	Clinical presentation	25	372	1372.4	5327.9	−0.385	0.046
NT-PRO BNP, P	pg/mL	Days 4–6 Post-Dx	14	20	815.3	4388.8	−0.929	0.02
Nucleated RBC	/100 WBC	Days 13–15 Post-Dx	23	189	1.24	0.447	0.561	1.7E-03
O_2 _HB	%	Days 1–3 Post-Dx	13	242	88.6	95	−1.37	2.2E-03
O_2 _HB	%	Days 4–6 Post-Dx	32	90	92.1	93.7	−0.356	0.013
O_2 _HB	%	Days 7–9 Post-Dx	24	46	91.5	94.5	−0.701	3.3E-04
Osmolality, U	mOsm/kg	Pre-diagnosis	4	80	231.5	478.8	−1.509	0.044
Oxygen Content, Arterial	vol %	Days 4–6 Post-Dx	32	89	16	13.7	0.839	2.4E-03
Oxygen Saturation (%) in Arterial Blood	%	Clinical presentation	27	189	94.2	96.2	−0.52	3.1E-03
Oxygen Saturation (%) in Arterial Blood	%	Days 1–3 Post-Dx	31	216	94.3	97.1	−1.293	8.4E-09
Oxygen Saturation (%) in Arterial Blood	%	Days 4–6 Post-Dx	26	70	94.3	95.7	−0.578	0.014
Oxygen Saturation (%) in Arterial Blood	%	Days 10–12 Post-Dx	18	29	93.4	96.5	−1.254	3.1E-03
Oxygen Saturation (%) in Arterial Blood	%	Days 13–15 Post-Dx	17	28	94.8	96.4	−0.671	0.043
pH Blood Arterial	None	Days 1–3 Post-Dx	26	193	7.42	7.39	0.539	0.035
pH Blood Venous	pH	Days 1–3 Post-Dx	10	82	7.42	7.36	0.963	0.031
pH, POCT, B	None	Clinical presentation	13	202	7.41	7.33	0.708	0.042
Platelets	×10(9)/L	Pre-diagnosis	39	649	184.8	225.9	−0.393	0.024
PO_2_	mm Hg	Days 1–3 Post-Dx	8	145	67.2	179.7	−1.301	1.7E-03
PO_2_	mm Hg	Days 7–9 Post-Dx	14	16	71.1	121.1	−0.949	0.027
PO_2_ Arterial	mm Hg	Days 1–3 Post-Dx	26	193	100.4	150.9	−0.87	8.2E-05
PO_2_ Arterial	mm Hg	Days 10–12 Post-Dx	17	25	93.6	134	−0.755	0.019
Potassium, S	mmol/L	Pre-diagnosis	10	398	3.93	4.35	−0.836	0.049
RABG Calculated O_2_ Hemoglobin	%	Days 1–3 Post-Dx	22	109	93.6	95	−0.464	2.9E-03
RABG Calculated O_2_ Hemoglobin	%	Days 4–6 Post-Dx	16	49	93.2	95.3	−0.859	2.3E-03
RABG Calculated O_2_ Hemoglobin	%	Days 10–12 Post-Dx	13	22	94	96.3	−1.269	0.038
RABG PF Ratio	None	Days 4–6 Post-Dx	17	49	1.46	2.68	−1.489	6.9E-05
RABG PF Ratio	None	Days 7–9 Post-Dx	13	22	1.75	2.56	−1.006	0.038
RABG PF Ratio	None	Days 10–12 Post-Dx	13	22	1.83	3.22	−1.518	3.9E-03
RBC (Red Blood Cell) Count	×10(12)/L	Clinical presentation	151	1671	4.32	3.99	0.409	2.0E-04
RBC (Red Blood Cell) Count	×10(12)/L	Days 1–3 Post-Dx	158	1562	4.13	3.73	0.524	5.8E-08
RBC (Red Blood Cell) Count	×10(12)/L	Days 4–6 Post-Dx	152	846	4.08	3.55	0.693	3.2E-12
RBC (Red Blood Cell) Count	×10(12)/L	Days 7–9 Post-Dx	132	635	4	3.49	0.656	2.4E-09
RBC (Red Blood Cell) Count	×10(12)/L	Days 10–12 Post-Dx	110	502	3.95	3.48	0.587	6.1E-07
Red Cell Distribution Width CV	%	Days 4–6 Post-Dx	137	722	14.1	15.1	−0.373	3.4E-04
Red Cell Distribution Width CV	%	Days 7–9 Post-Dx	119	552	14.2	15.4	−0.431	9.8E-05
Red Cell Distribution Width CV	%	Days 10–12 Post-Dx	97	429	14.5	15.7	−0.394	1.2E-03
Sodium, P	mmol/L	Clinical presentation	89	1141	135.6	137.3	−0.375	7.3E-03
Sodium, P	mmol/L	Days 1–3 Post-Dx	77	927	136.6	138.1	−0.377	4.7E-03
Sodium, S	mmol/L	Days 10–12 Post-Dx	69	334	140.8	138.3	0.651	2.0E-04
Spont. Breaths/min	None	Days 4–6 Post-Dx	23	67	25	20.2	0.767	0.016
Tacrolimus, B	ng/mL	Days 7–9 Post-Dx	8	81	4.22	8.12	−1.102	8.8E-03
Tacrolimus, B	ng/mL	Days 10–12 Post-Dx	8	79	3.8	9.24	−1.468	2.5E-03
Tacrolimus, B	ng/mL	Days 13–15 Post-Dx	7	71	3.7	8.52	−1.47	7.5E-03
Tacrolimus, B	ng/mL	Days 16–30 Post-Dx	10	110	4.93	7.8	−1.094	0.022
Temperature	None	Clinical presentation	23	136	37	36.7	0.591	0.042
Temperature	None	Days 1–3 Post-Dx	23	189	37	36.4	0.765	4.8E-04
Triglycerides, S/P	mg/dL	Days 4–6 Post-Dx	16	41	326.2	173	1.196	7.3E-03
Triglycerides, S/P	mg/dL	Days 7–9 Post-Dx	17	24	310.6	191.5	0.945	0.016
Triglycerides, S/P	mg/dL	Days 10–12 Post-Dx	17	35	364.5	174.4	1.217	4.0E-03
Triglycerides, S/P	mg/dL	Days 16–30 Post-Dx	10	77	276.1	166.4	0.83	0.024
Troponin T, 5TH GEN, P	ng/L	Days 4–6 Post-Dx	18	54	21.4	245.3	−0.499	7.5E-03
Troponin T, Baseline, 5TH Gen, P	ng/L	Days 7–9 Post-Dx	11	43	15.1	53.7	−0.538	0.037
VBGRS HGB	g/dL	Days 4–6 Post-Dx	36	99	12.3	10.5	0.932	3.6E-04
White Blood Cells	×10(9)/L	Days 1–3 Post-Dx	158	1650	6.67	9.08	−0.439	3.2E-12

With respect to coagulation, we found that plasma fibrinogen was significantly elevated in COVID_pos_ patients at the time of diagnosis (Cohen’s D = 0.859, BH-adjusted Mann-Whitney p-value = 8.9e-7, Table 2, Figure 3A). This hyperfibrinogenemia generally resolved during the 7 days following diagnosis (Figure 3A). Conversely, platelet counts were lower in the COVID_pos_ cohort at the time of clinical presentation but tended to increase over the subsequent 10 days to levels significantly higher than those in COVID_neg_ patients (Cohen’s D = 0.229, BH-adjusted Mann-Whitney p-value = 3.6e-3, Table 2, Figure 3B). While thrombocytopenia has been reported in COVID-19 patients before (Xu et al., 2020; Yang et al., 2020), an upward trend in platelet counts after diagnosis has not been described to our knowledge. We observe extended prothrombin times in both the COVID_pos_ and COVID_neg_ (matched) cohorts significantly above the normal range; however, there was no differentiation between the cohorts. We observe extended activated partial thromboplastin times (aPTT) in the COVID_pos_ significantly above normal levels from day 7 onward (Figure 3D). D-dimer levels were frequently above normal limits in both the COVID_pos_ and COVID_neg_ cohorts and were not significantly different between these cohorts during any time window (Figure 3E). The above trends hold up even when the time windows are perturbed (Table 3).

**Table 3.** Sensitivity analysis of clinical time intervals for significant coagulation-related lab test trends. Results from sensitivity analysis perturbing the time intervals for the significant (coagulation-related lab test, time interval) pairs (i.e. highlighted rows of Table 2). Perturbed results that met both of the significance thresholds (BH-adjusted Mann-Whitney p-value <0.05 and Cohen’s D absolute value >0.35) are highlighted in light green, and perturbed results that only met one of the thresholds for either effect size or statistical significance are highlighted in yellow.

Test	Units	Perturbation	Original time window	Count COVID_pos_	Count COVID_neg_	Mean COVID_pos_	Mean COVID_neg_	Cohen's D	BH-adjusted M-W p-value
Activated Partial Thrombopl Time, P	sec	−1 day	Days 7−9 Post-Dx	26	72	50.1	38	0.57	0.034
Activated Partial Thrombopl Time, P	sec	+1 day	Days 7−9 Post-Dx	17	58	55	37.5	0.81	0.014
Activated Partial Thrombopl Time, P	sec	−1 day	Days 10−12 Post-Dx	16	57	56.9	38.4	0.808	9.10E-03
Activated Partial Thrombopl Time, P	sec	+1 day	Days 10−12 Post-Dx	15	60	56.9	38	1.106	2.60E-03
Activated Partial Thrombopl Time, P	sec	−1 day	Days 13−15 Post-Dx	15	52	55.5	37.8	1.041	0.014
Activated Partial Thrombopl TIME, P	sec	+1 day	Days 13−15 Post-Dx	14	48	51.8	37.1	0.962	0.015
Activated Partial Thrombopl Time, P	sec	−1 day	Days 16−30 Post-Dx	22	156	55.2	37	0.913	5.70E-03
Activated Partial Thrombopl Time, P	sec	+1 day	Days 16−30 Post-Dx	19	139	56	38.2	0.725	3.80E-02
Fibrinogen, P	mg/dL	−1 day	Clinical presentation	25	92	584.9	370.7	1.067	1.20E-04
Fibrinogen, P	mg/dL	+1 day	Clinical presentation	37	292	488.2	326.2	0.885	8.80E-06
Fibrinogen, P	mg/dL	−1 day	Days 1−3 Post-Dx	41	381	494.5	318	1.023	3.90E-07
Fibrinogen, P	mg/dL	+1 day	Days 1−3 Post-Dx	21	244	420.3	312.2	0.616	7.90E-03
Fibrinogen, P	mg/dL	−1 day	Days 4−6 Post-Dx	27	156	432.2	336	0.495	0.045
Fibrinogen, P	mg/dL	+1 day	Days 4−6 Post-Dx	24	105	472.2	333.2	0.712	0.025
Platelets	x10(9)/L	−1 day	Pre-diagnosis	34	575	187.3	225.6	-0.357	0.057
Platelets	x10(9)/L	+1 day	Pre-diagnosis	118	1533	201.3	234.4	-0.328	7.30E-04

**Figure 4.**
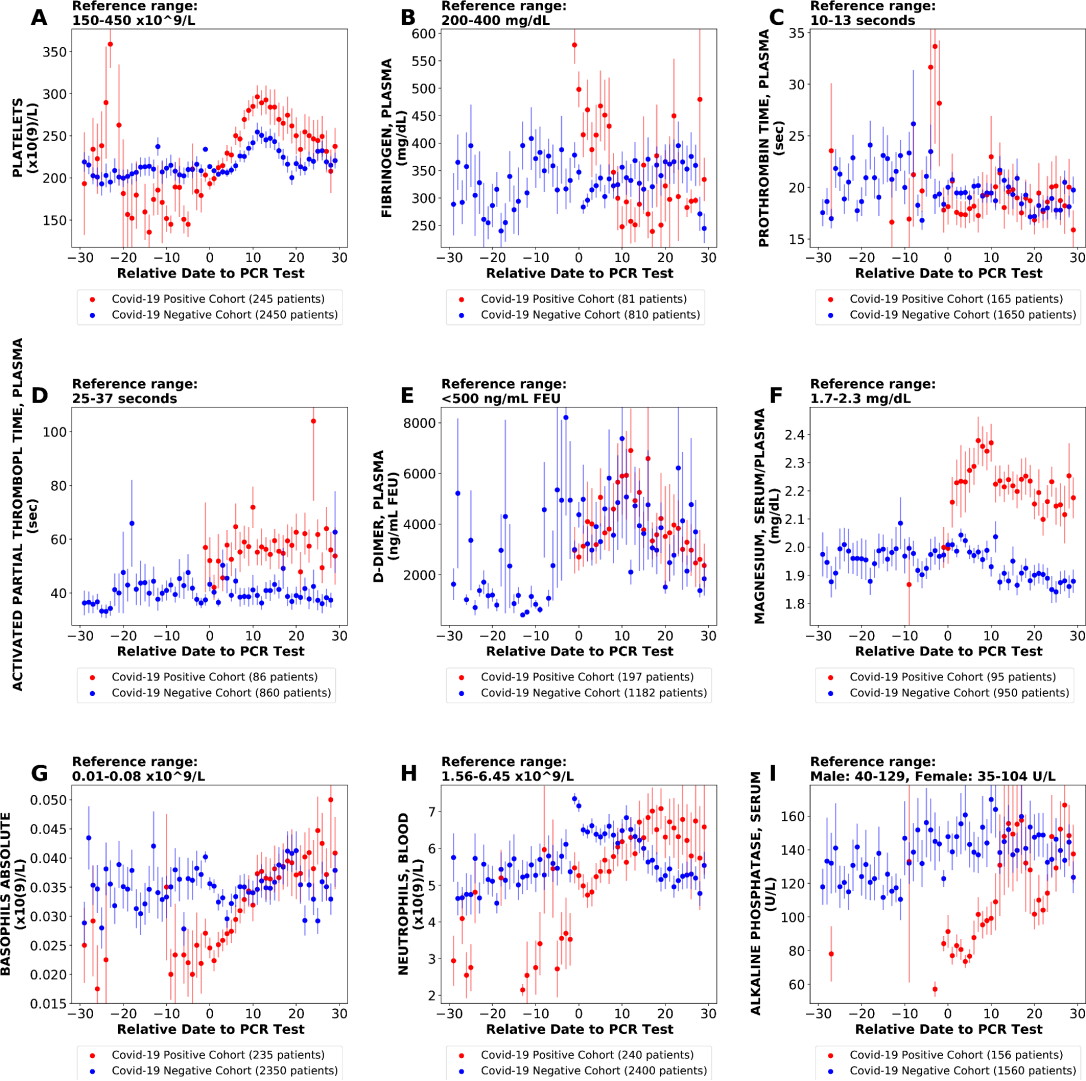
Longitudinal trends of lab tests with daily resolution. Longitudinal trends of COVID_pos_ versus COVID_neg_ (matched) patients for the following lab tests: (**A**) platelets; (**B**) fibrinogen, plasma; (**C**) prothrombin time, plasma; (**D**) activated partial thromboplastin time; (**E**) D-dimer; (**F**) magnesium, serum/plasma; (**G**) basophils absolute; (**H**) neutrophils, blood; (**I**) alkaline phosphatase, serum. The reference ranges are shown at the top of each plot. For each cohort, average lab values and standard errors are shown for each day with at least three observations. For certain lab tests, some data points are missing because these days had fewer than three data points in the COVID_pos_ cohort.

We also performed similar analyses comparing the COVID_pos_ and COVID_neg_ (matched) cohorts using different time window definitions including daily trends (Figure 4). This approach offers the advantage of increased granularity at the cost of sample size per time point, but we did identify similar lab tests as altered in COVID_pos_ patients using each approach including the fibrinogen decline and platelet increase in the COVID_pos_ cohort after diagnosis (Figure 4).

### Thrombosis is enriched among COVID-19 patients undergoing longitudinal lab testing

Given the recently described coagulopathies associated with COVID-19 (Tang et al., 2020; Klok et al., 2020; Levi et al., 2020), we were intrigued by the temporal trends in fibrinogen levels and platelet counts in the COVID_pos_ cohort (Figure 3). Next, we asked whether the observed coagulation-related laboratory trends were associated with clinical manifestations of thrombosis. To do so, we employed a BERT-based neural network (Devlin et al., 2018; see Materials and methods) to identify patients who experienced a thrombotic event after their SARS-CoV-2 PCR testing date. Specifically, we extracted diagnostic sentiment from EHR notes (e.g. whether a patient was diagnosed with a phenotype, suspected of having a phenotype, ruled out for having a phenotype, or other) regarding specific thromboembolic phenotypes including deep vein thrombosis, pulmonary embolism, myocardial infarction, venous thromboembolism, thrombotic stroke, cerebral venous thrombosis, and disseminated intravascular coagulation.

We found that 101 of the total 2232 COVID_pos_ cohort (4.5%) were positively diagnosed with one or more of the above-mentioned thrombotic phenotypes in the 30 days after PCR testing, with the majority of these patients (53 of 101) experiencing a deep vein thrombosis. Interestingly, we found that after creating subsets of the patients with longitudinal lab testing data (i.e. the patients meeting the criteria for inclusion in our study), 76 of the 246 patients (31%) had at least one EHR-derived clot diagnosis, including 47 patients with deep vein thrombosis (Table 4). Thus, the cohort under consideration here is highly enriched (Table 5; hypergeometric p-value <1×10^−50^) for patients experiencing thrombotic events compared to the overall COVID_pos_ cohort.

**Table 4.** Prevalence of thrombotic phenotypes after the clinical presentation in COVID_pos_ patients with and without available longitudinal lab testing data. For each clotting phenotype listed, a BERT-based neural network was used to extract diagnostic sentiment from individual EHR patient notes in which the phenotype (or a synonym thereof) was present. This automated curation was applied to clinical notes for each patient from day = −1 (clinical presentation) to day = 30 (end of the study period) relative to the PCR testing date. In this table, we show the absolute number of patients with each phenotype along with the percentage of patients in each cohort with the given specific thrombotic phenotype in parentheses.

Clotting phenotype	Cohort 1: COVID_pos_ with longitudinal data	Cohort 2: COVID_pos_ without longitudinal data	Cohort 3: Complete COVID_pos_ cohort
Deep vein thrombosis	47 (19%)	6 (0.30%)	53 (2.4%)
Pulmonary embolism	22 (8.9%)	9 (0.45%)	31 (1.4%)
Myocardial infarction	10 (4.1%)	8 (0.40%)	18 (0.81%)
Venous thromboembolism	7 (2.8%)	0	7 (0.31%)
Thrombotic stroke	2 (0.81%)	2 (0.10%]	4 (0.18%)
Cerebral venous thrombosis	0	0	0
Disseminated intravascular coagulation	5 (2.0%)	0	5 (0.22%)
Total unique patients with clot	76 (31%)	25 (1.3%)	101 (4.5%)
Total patients	246	1986	2232

**Table 5.** Enrichment of thrombotic phenotypes among COVID_pos_ patients with longitudinal lab testing data. Contingency table to calculate hypergeometric enrichment significance of thrombosis among patients with longitudinal lab testing data. The 246 patients with longitudinal testing data are those considered in this study, while the 1986 patients who did not have at least three results from one lab test over the defined 60-day window were excluded from this longitudinal analysis.

	Patient has longitudinal data	Patient does NOT have longitudinal data	Total
Thrombosis	76	25	101
No thrombosis	170	1961	2131
Total	246	1986	2232

Hypergeometric enrichment: p-value <1×10^−50^.

**Table 6.** Validation of the BERT model to identify the sentiment of thrombotic phenotypes in clinical notes. Out-of-sample accuracy results of the BERT model to identify thrombotic phenotypes in 1000 randomly selected sentences from clinical notes which contained at least one mention of a thrombotic phenotype. The columns are (1) *Clotting phenotype:* thrombotic phenotype identified in the sentence, (2) *TP (true positives):* count of sentences in which the BERT model correctly identified the sentiment as ‘Yes’, (3) *TN (true negatives):* count of sentences in which the BERT model correctly identified the sentiment as not ‘Yes’, (4) *FP (false positives):* count of sentences in which the BERT model incorrectly identified the sentiment as ‘Yes’, (5) *FN: (false negatives):* count of sentences in which the BERT model incorrectly identified the sentiment as not ‘Yes’, (6) *Recall:* recall of the BERT model, equal to TP/(TP+FN), (7) *Precision:* precision of the BERT model, equal to TP/(TP+FP), (8) *Accuracy:* accuracy of the BERT model, equal to (TP+TN)/(TP+TN+FP+FN).

Clotting phenotype	TP	TN	FP	FN	Recall	Precision	Accuracy
Deep vein thrombosis	136	178	24	3	98%	85%	92%
Pulmonary embolism	164	78	7	6	96%	96%	95%
Myocardial infarction	212	65	3	3	99%	99%	98%
Venous thromboembolism	3	97	7	0	100%	30%	93%
Thrombotic stroke	5	0	0	0	100%	100%	100%
Cerebral venous thrombosis	1	0	0	0	100%	100%	100%
Disseminated intravascular coagulation	4	4	0	0	100%	100%	100%
Overall	525	422	41	12	97.8%	92.8%	94.7%

### Longitudinal platelet count trends are not strongly associated with the development of thrombosis in COVID-19 patients

**Figure 5.**
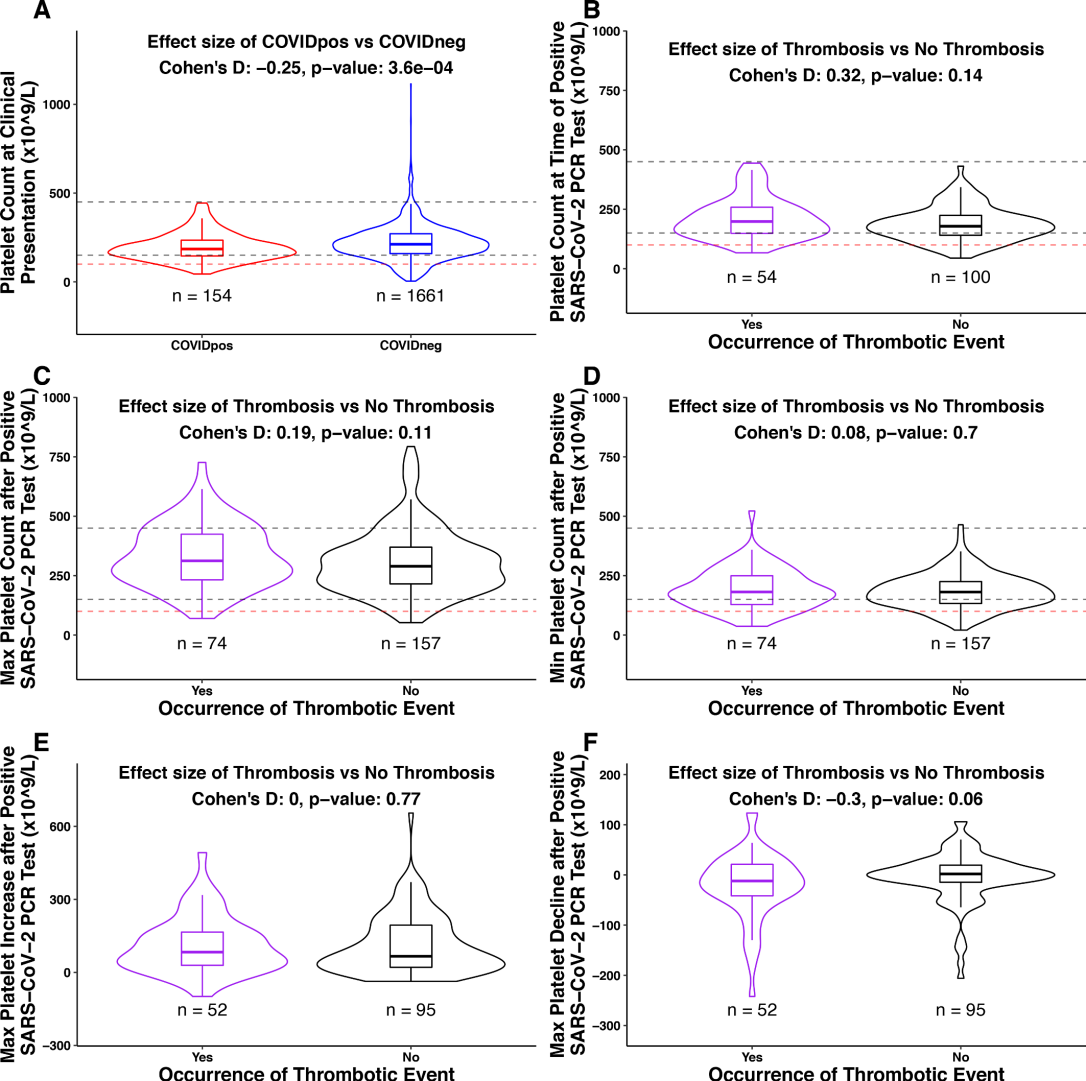
Association between platelet counts and thrombosis in the COVID_pos_ cohort. Box plots of platelet counts, min/max values, and maximum levels of increase/decline at specific time intervals for COVID_pos_ and COVID_neg_ cohorts and subgroups of the COVID_pos_ cohort with and without thrombotic events after SARS-CoV-2 diagnosis. In the subplot (**A**), we show platelet counts for COVID_pos_ (red) and COVID_neg_ (blue) cohorts. In subplots (**B-F**), we show platelet counts for COVID_pos_ patients who did and did not subsequently develop thromboses (purple and black, respectively). Horizontal dotted gray lines correspond to upper and lower limits of normal platelet counts (150−450 × 10^9^/L), and horizontal red line shows 100 × 10^9^/L. At the top of each plot, Cohen’s D effect size and p-value from the Mann-Whitney statistical test are shown. (**A**) Platelet counts at the time of PCR testing for COVID_pos_ and COVID_neg_ cohorts. (**B**) Platelet counts at the time of PCR testing for COVID_pos_ patients who did and did not subsequently develop thromboses. (**C**) Maximum platelet counts (considering counts at and after positive PCR test date) for COVID_pos_ patients who did and did not subsequently develop thromboses. (**D**) Minimum platelet counts (considering counts at and after positive PCR test date) for COVID_pos_ patients who did and did not subsequently develop thromboses. (**E**) Maximum degree of platelet increases after positive PCR test date for COVID_pos_ patients who did and did not subsequently develop thromboses. (**F**) Maximum degree of platelet declines after positive PCR test date for COVID_pos_ patients who did and did not subsequently develop thromboses.

Among the 246 COVID_pos_ patients with longitudinal lab testing data, 81 were serially tested starting at clinical presentation for fibrinogen versus 245 tested for platelets. As such, we first analyzed whether associations exist between platelet counts (or temporal alterations thereof) and clotting propensity in this cohort. Among these 245, there were 169 patients without thrombosis after PCR-based diagnosis (non-thrombotic) and 76 patients with thrombosis (thrombotic). There is a statistically significant difference between the COVID_pos_ and COVID_neg_ cohorts in the platelet count at clinical presentation (Figure 5A). In particular, thrombocytopenia (platelet count <150×10^9^/L) was observed in 29% (46 out of 154) COVID_pos_ and 21% (346 of 1661) COVID_neg_ patients at the time of diagnosis (Figure 5A). However, the platelet levels at this time point were not associated with the subsequent formation of a blood clot in the COVID_pos_ cohort (Figure 5B).

We hypothesized that the previously discussed increase in platelet counts after COVID-19 diagnosis may be associated with the development of blood clots. If true, then we would expect the thrombotic COVID_pos_ cohort to show significantly higher maximum platelet counts during their course of disease progression compared to the non-thrombotic COVID_pos_ cohort. We found that this was not the case, as maximum platelet counts were similar in the two groups (Figure 5C). Similarly, among the 147 COVID_pos_ patients with platelet counts both at the time of clinical presentation and post-diagnosis, the degree of maximal platelet increase was not associated with the development of thrombosis (Figure 5E). It would certainly be of interest to perform this same analysis on a larger COVID_pos_ cohort (n = 2232; 101 thrombotic vs. 2131 non-thrombotic), but we were not able to do so given the lack of longitudinal testing available for a large majority of non-thrombotic COVID_pos_ patients (Table 4).

Conversely, we explored whether some COVID_pos_ patients may experience clotting in the setting of low or declining platelets (e.g. consumptive coagulopathy) despite the population-level trend of increasing platelets over time. Indeed, we found that nine of 74 thrombotic patients showed absolute platelet counts below 100 × 10^9^/L during at least one post-diagnosis time window (below dotted red line in Figure 5D). In addition, we analyzed post-diagnosis platelet reductions among COVID_pos_ patients. While the maximum degree of absolute platelet reduction was not associated with clot development in aggregate (Figure 5F), we did find that six of the 52 thrombotic patients experienced a reduction of at least 100 × 10^9^/L relative to the time of diagnosis. Of note, similar fractions of non-thrombotic COVID_pos_ patients also showed these low or declining platelet counts, indicating that these trends are not specific indicators of thrombosis (Figure 5D,F).

### Consumptive coagulopathy contributes to only a small fraction of COVID-19 associated thromboses

The observed declining platelet counts and thrombocytopenia in the context of thrombosis in a small fraction of COVID_pos_ patients are consistent with previous reports that fewer than 1% of survivors, but over 70% of non-survivors, meet the International Society on Thrombosis and Hemostasis (ISTH) criteria for disseminated intravascular coagulation (DIC; Tang et al., 2020). As was previously noted, hyperfibrinogenemia was among the strongest lab test features distinguishing COVID_pos_ from COVID_neg_ patients at diagnosis, but the subsequent downward trend (Figure 3A) could be attributed to a resolving acute phase response and/or consumption of fibrinogen in a systemic coagulopathy. Using our BERT-based sentiment extraction, we found that only five of the 2232 COVID_pos_ patients that exhibited DIC-like symptoms, all of whom were included in our longitudinal cohort of 246 COVID_pos_ patients (Table 4). Upon manual review of the EHR data for each patient, we found that two out of these five patients had confirmed diagnosis of DIC, while the remaining had high clinical suspicion and pending tests for DIC. This finding suggests that declining fibrinogen after COVID-19 diagnosis typically represents a physiologic return to normal range rather than pathologic coagulation factor consumption. To further examine the plasma fibrinogen trends among COVID-19 patients with DIC, with non-DIC thrombosis, and without thrombosis, we examined patient-level lab test trends from 10 individuals who were tested for fibrinogen both at the time of diagnosis and at least two times subsequently. The 10 patients for individual analysis were selected as the first 10 individuals with longitudinal fibrinogen lab testing data available.

**Figure 6.**
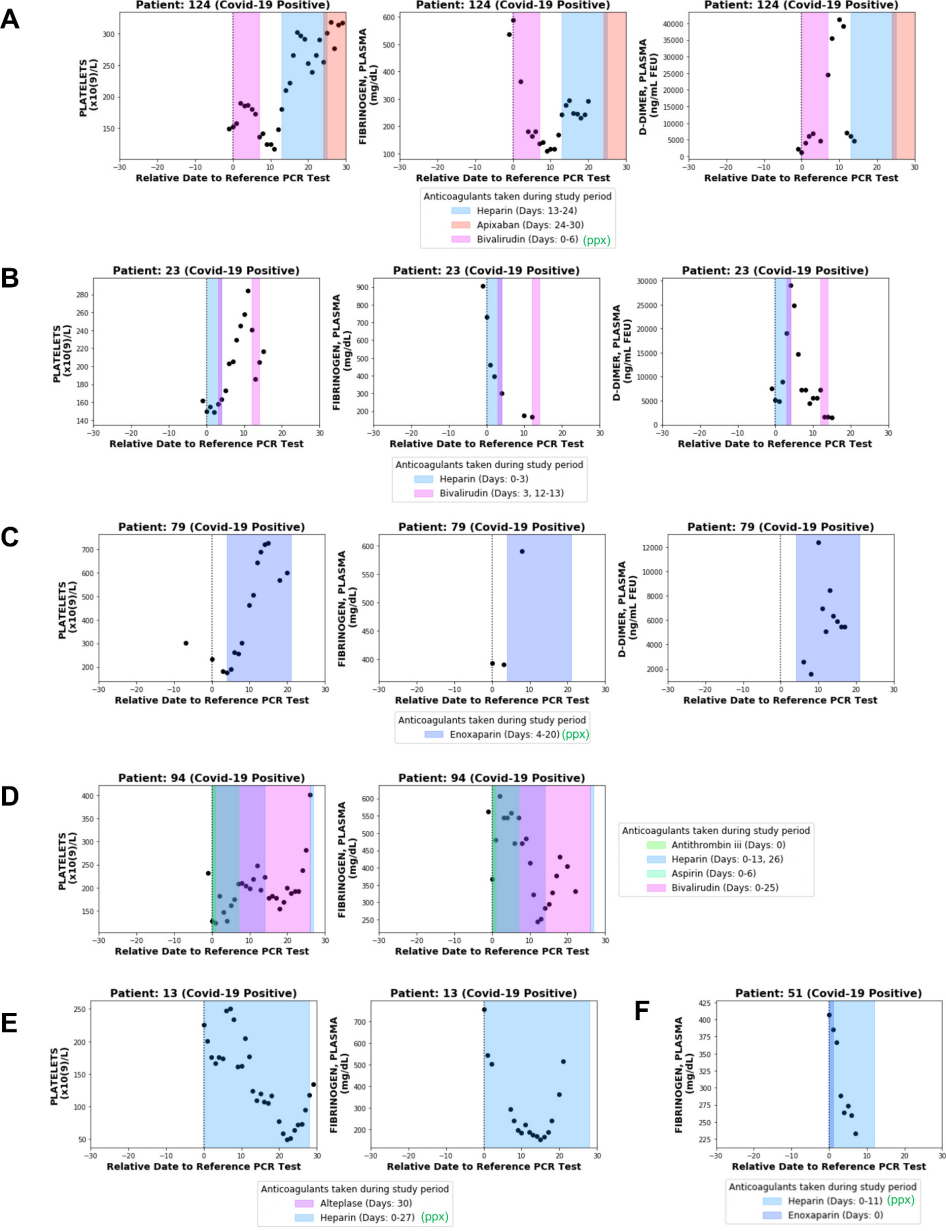
Longitudinal analyses of platelet counts, plasma fibrinogen, and D-dimer levels in individual patients with or without thrombotic disease. In each plot, shaded regions represent time periods when the patient was taking a specific anticoagulant or antiplatelet medication. Medications taken for prophylaxis are denoted in the legend with (ppx). (**A**) Patient 124 developed hemorrhagic and thrombotic phenotypes in the context of declining fibrinogen, declining platelets, and increasing D-dimers. This is consistent with a DIC-like coagulopathy. (**B**) Patient 23 developed clots in the setting of declining fibrinogen and elevated D-dimers but stable platelet counts which increased shortly thereafter. (**C**) Patient 79 developed clots while showing increases in platelet counts along with plasma fibrinogen and D-dimers. (**D**) Patient 94 developed clots with relatively stable platelet counts and steadily declining plasma fibrinogen. (**E**) Patient 13 did not develop clots or bleeding despite a coordinate decrease in platelet counts and fibrinogen which may be mistaken for a DIC-like coagulopathy. (**F**) Patient 51 did not develop clots despite showing a post-diagnosis decline in plasma fibrinogen similar to several patients in the thrombotic cohort.

This patient-level analysis indeed revealed multiple distinct trajectories with respect to fibrinogen and other coagulation parameters in COVID_pos_ patients. Four of these ten individuals developed at least one blood clot during their hospital course. Only one was identified by our BERT model (and confirmed by manual EHR review) to have low-grade DIC, and as expected we found this patient’s longitudinal lab test pattern to be consistent with consumptive coagulopathy (Patient 124; Figure 6A). At the time of diagnosis, this patient showed significant hyperfibrinogenemia with elevated D-dimers (1304.5 ng/mL) and a borderline normal platelet count (153 × 10^9^/L). Over the next 10 days, this patient’s fibrinogen levels consistently decreased, reaching a minimum of 110 mg/dL on day 9. Similarly, after an initial recovery to 190 × 10^9^/L the platelet counts consistently declined starting on day 2 post-diagnosis, reaching a minimum of 117 × 10^9^/L on day 11. D-dimer levels exponentially increased after 5 days, reaching a maximum of 41,300 ng/mL on day 10. Phenotypically, this patient experienced both thrombotic (right internal jugular vein and right superior thyroid artery) and hemorrhagic (oropharyngeal and pulmonary) events. This combination of lab results and clinical manifestations is consistent with the diagnosis of DIC-like consumptive coagulopathy during the first week after COVID-19 diagnosis.

Lab test results from three other non-DIC thrombotic patients with longitudinal fibrinogen testing confirm the presence of alternative forms of coagulopathy in the COVID-19 population. Patient 23 developed a clot on day 4 post-diagnosis in the context of a declining fibrinogen level and increasing D-dimers but steady platelet counts, which actually increased shortly thereafter (Figure 6B). Patient 79 developed several clots after day 3 post-diagnosis in the setting of upward trending platelets (which eventually exceed the upper limit of normal) and elevated levels of both fibrinogen and D-dimers (Figure 6C). Patient 94 developed a clot on day 8 post-diagnosis with relatively stable platelet counts within normal limits and steadily declining fibrinogen levels (Figure 6D).

One hypothesis is that early elevations in plasma fibrinogen contribute to the clotting observed in the non-DIC like COVID_pos_ cohort. This hypothesis may warrant further analysis in cohorts with more longitudinal fibrinogen data, but again it is important to note that several COVID_pos_ patients who presented with hyperfibrinogenemia did not go on to develop thromboses (Figure 6E–F). This emphasizes that a steady post-diagnosis decline in plasma fibrinogen may represent physiologic resolution of the acute phase response rather than a pathologic consumption of fibrinogen and other coagulation factors (Figure 6B,D–F).

Taken together, this analysis affirms that a DIC-like coagulopathy resulting in a combination of hemorrhage and thrombosis can develop in the setting of COVID-19 infection. However, the observations that DIC was formally diagnosed in only five of 2232 COVID_pos_ patients and emphasizes that consumptive coagulopathy is an exception rather than the rule as it pertains to thrombotic phenotypes in COVID-19 patients. These results should be considered as a preliminary characterization of COVID-associated coagulopathies (CAC) and will be updated as patient counts increase with the continued evolution of the COVID-19 pandemic.

## Discussion

Many studies on clinical characteristics and lab tests are shedding light on the spectrum of hematological parameters associated with COVID-19 patients. In an initial study of 41 patients from Wuhan, the blood counts in COVID_pos_ patients showed leukopenia and lymphopenia, and prothrombin time and D-dimer levels were higher in ICU patients than in non-ICU patients (Huang et al., 2020). Another study based on 343 Wuhan COVID_pos_ patients found that a D‐dimer level of at least 2.0 µg/mL could predict mortality with a sensitivity of 92.3% and a specificity of 83.3% (Zhang et al., 2020). An independent study of 43 COVID-19 patients found significant differences between mild and severe cases in plasma interleukin‐6 (IL‐6), D‐dimers, glucose, thrombin time, fibrinogen, and C‐reactive protein (p<0.05; Gao et al., 2020). While such studies indeed highlight that hematological and inflammatory abnormalities are prevalent in COVID_pos_, a high-resolution temporal understanding of how these parameters evolve in COVID-19 patients post diagnosis has not been established. Specifically, in the wake of accumulating evidence for hypercoagulability in COVID_pos_ patients, there are important clinical questions emerging regarding the necessity of and guidelines for thromboprophylaxis in patient management.

DIC-like consumptive coagulopathy in COVID-19 has been a point of concern in severely ill COVID-19 patients. Particularly in patients with ARDS, multiple organ dysfunction syndrome (MODS) is the predominant cause of death. A recent study suggested that DIC was associated with MODS during the early stage of ARDS and that persistent DIC may also have a role in this association (Gando et al., 2020). Our study focusing on COVID-19 patients with longitudinal lab data suggests that COVID-19 is indeed associated with modulation of coagulation related parameters such as platelet counts, fibrinogen levels, and clotting time (Figure 2). However, the majority of thrombotic events in COVID-19 patients with longitudinal lab testing are not the result of a DIC-like consumptive coagulopathy, as this only occurs in a small subset (Table 4).

The ability to derive this longitudinal understanding of COVID-19 progression, including laboratory abnormalities and their associated clinical manifestations, mandates the synthesis of structured and unstructured EHR data (e.g. lab tests and clinical notes) at a large scale. The fact that tens of thousands of patients have undergone SARS-CoV-2 testing at major academic medical centers (AMCs) provides an abundance of potential data to perform this analysis but also poses significant challenges from a practicality standpoint. Manual review and curation of patient trajectories and associated testing results is not practical. It is not likely to provide comprehensive or even entirely accurate individual patient records. Rather, triangulation across datasets, including lab measurements, clinical notes, and prescription information, using a scalable digitized approach to extract structured data along with sentiment-surrounded clinical phenotypes and outcomes enables us to efficiently perform this analysis in a timely fashion.

By developing and deploying such a digitized platform on the entirety of EHR data from a large AMC, we have identified in an unbiased manner, laboratory test-based abnormalities that differentiate COVID_pos_ patients from COVID_neg_ patients. The abnormalities in coagulation-related tests, including fibrinogen and platelets, were intriguing in the context of literature reporting the occurrence of various clotting phenotypes in COVID-19 patients, including DIC-like consumptive coagulopathies along with more isolated clotting events in the lungs, central nervous system, and other tissues (Tang et al., 2020; Klok et al., 2020; Levi et al., 2020). Our finding that consumptive coagulopathy represents a minority of COVID-19 associated clotting events provides context for other studies, which have reported overt DIC or DIC-like disease in over 70% of non-survivors but far lower fractions of survivors (Tang et al., 2020). As the pandemic continues to evolve and the patient counts increase over the coming months, we will be monitoring and reporting any updates to the clinical and laboratory observations drawn in this study.

Notwithstanding the preliminary nature of the analysis presented in this study, the results highlight that consumptive coagulopathy should be considered in the minority of COVID_pos_ patients with significant serial reductions in platelet counts. It remains to be seen whether the post-diagnosis platelet increases or early hyperfibrinogenemia which we observed may contribute mechanistically to the clotting in the much larger non-DIC thrombotic COVID-19 population. It is important to note that despite the trend of increasing platelets, the platelet count only extended above the normal range (>450×10^9^/L) after the PCR date in few COVID_pos_ patients with serial measurements, and the development of such outright thrombocytosis was observed with similar frequencies in the thrombotic and non-thrombotic cohorts (Figure 5C). Further, the fact that several patients with elevated fibrinogen (i.e. >400 mg/dL) at presentation did not develop thromboses suggests that early hyperfibrinogenemia is not a singular driver of subsequent clotting events, but a small sample size (n = 10 patients; nine non-thrombotic vs. one thrombotic) limited the power of this analysis (Figure 6).

Despite these caveats, this linking of longitudinal trends to patient outcomes provides several useful pieces of clinical information. First, hyperfibrinogenemia is to be expected in COVID-19 patients around the time of diagnostic testing. Furthermore, declining fibrinogen levels shortly after diagnosis are also expected and likely represent the resolution of acute phase response in most patients rather than a decline secondary to the onset of consumptive coagulation. In addition, borderline or overt thrombocytopenia is common in COVID-19 patients at the time of clinical presentation, and the initial platelet count does not robustly predict patients who are likely to develop thromboses. After diagnosis, COVID-19 patients generally show an upward trend in platelets. Patients whose platelets trend down after diagnosis should be monitored, as platelet reductions after clinical presentation are associated with thromboses and significant reductions may be indicative of ongoing consumptive coagulopathy.

One unavoidable limitation of this study is that we restrict our analysis to patients which have longitudinal lab testing data available. While the inclusion criteria is naturally biased, we consider this study population to be of high clinical interest because these patients are highly enriched for severe thrombotic events during the study period (see Table 5). Further, in the propensity score matching step of the analysis, we are able to construct a control cohort that is similar to the COVID_pos_ cohort in these enriched dimensions. To provide additional color on the distinctive attributes of the study population, we provide a summary of the clinical characteristics of the study population versus all patients with PCR tests during the same time period (see Table 7). In addition, we provide the median numbers of lab tests per patient for selected coagulation-related lab tests (fibrinogen, platelets, PTT, APTT, D-dimer) and total lab tests (Tables 8 and 9).

**Table 7.** General characteristics of patients with SARS-CoV-2 PCR testing. General demographic characteristics of all patients who underwent SARS-CoV-2 PCR testing in the Mayo Clinic EHR database from February 15, 2020 to May 28, 2020. Includes summary characteristics for: (A) all patients with at least one SARS-CoV-2 PCR test, and (B) patients with at least one SARS-CoV-2 PCR test and longitudinal testing data available (i.e. patient received the same lab test on 3 separate days within + / − 30 days of PCR testing date).

(A) Demographics of all patients with PCR testing data		
	COVID_pos_	COVID_neg_
Total number of patients	2232	72,354
Gender:		
Male	1153 (52%)	31,613 (44%)
Female	1074 (48%)	40,714 (56%)
Race:		
White	1115 (50%)	62,605 (87%)
Black	420 (19%)	2792 (3.9%)
Asian	151 (6.8%)	1719 (2.4%)
American Indian	29 (1.3%)	302 (0.42%)
Other	517 (23%)	4936 (6.8%)
(B) Demographics of patients with PCR testing data and longitudinal testing data									
Test	Units	Perturbation	Original time window	Count COVID_pos_	Count COVID_neg_	Mean COVID_pos_	Mean COVID_neg_	Cohen's D	BH-adjusted M-W p-value
Activated Partial Thrombopl Time, P	sec	−1 day	Days 7–9 Post-Dx	26	72	50.1	38	0.57	0.034
Activated Partial Thrombopl Time, P	sec	+1 day	Days 7–9 Post-Dx	17	58	55	37.5	0.81	0.014
Activated Partial Thrombopl Time, P	sec	−1 day	Days 10–12 Post-Dx	16	57	56.9	38.4	0.808	9.10E-03
Activated Partial Thrombopl Time, P	sec	+1 day	Days 10–12 Post-Dx	15	60	56.9	38	1.106	2.60E-03
Activated Partial Thrombopl Time, P	sec	−1 day	Days 13–15 Post-Dx	15	52	55.5	37.8	1.041	0.014
Activated Partial Thrombopl Time, P	sec	+1 day	Days 13–15 Post-Dx	14	48	51.8	37.1	0.962	0.015
Activated Partial Thrombopl Time, P	sec	−1 day	Days 16–30 Post-Dx	22	156	55.2	37	0.913	5.70E-03
Activated Partial Thrombopl Time, P	sec	+1 day	Days 16–30 Post-Dx	19	139	56	38.2	0.725	3.80E-02
Fibrinogen, P	mg/dL	−1 day	Clinical presentation	25	92	584.9	370.7	1.067	1.20E-04
Fibrinogen, P	mg/dL	+1 day	Clinical presentation	37	292	488.2	326.2	0.885	8.80E-06
Fibrinogen, P	mg/dL	−1 day	Days 1–3 Post-Dx	41	381	494.5	318	1.023	3.90E-07
Fibrinogen, P	mg/dL	+1 day	Days 1–3 Post-Dx	21	244	420.3	312.2	0.616	7.90E-03
Fibrinogen, P	mg/dL	−1 day	Days 4–6 Post-Dx	27	156	432.2	336	0.495	0.045
Fibrinogen, P	mg/dL	+1 day	Days 4–6 Post-Dx	24	105	472.2	333.2	0.712	0.025
Platelets	×10(9)/L	−1 day	Pre-diagnosis	34	575	187.3	225.6	−0.357	0.057
Platelets	×10(9)/L	+1 day	Pre-diagnosis	118	1533	201.3	234.4	−0.328	7.30E-04

**Table 8.** Lab test data availability in patients with SARS-CoV-2 PCR testing. Lab test data availability for all patients who underwent SARS-CoV-2 PCR testing in the Mayo Clinic EHR database from February 15, 2020 to May 28, 2020. Includes counts of lab tests and counts of patients with 1+ and 3+ lab tests both overall and for selected coagulation-related lab tests (activated partial thromboplastin time, D-dimer, fibrinogen, platelets, and prothrombin time).

	COVID_pos_	COVID_neg_
Total number of patients	2232	72,354
Number of patients with 1+ lab test	566 (25%)	35,188 (49%)
Number patents with 1+ test from day −30 to day −1	299 (13%)	23,116 (32%)
Number patents with 1+ test from day 0 to day 30	452 (20%)	28,666 (40%)
Number of patients with 3+ lab tests of the same type	246 (11%)	13,666 (19%)
Total number of lab tests	98,753	32,40,491
Number of lab tests from day −30 to day −1	12,120	10,33,762
Number of lab tests from day 0 to day 30	86,633	22,06,729
ACTIVATED PTT		
Number of lab tests	362	6042
Number of patients with 1+ lab test	93 (4.0%)	3544 (4.9%)
Number of patients with 3+ lab tests	20 (0.86%)	406 (0.56%)
D-DIMER, P		
Number of lab tests	911	2846
Number of patients with 1+ lab test	247 (11%)	2395 (3.3%)
Number of patients with 3+ lab tests	99 (4.4%)	56 (0.077%)
FIBRINOGEN, P		
Number of lab tests	278	3,017
Number of patients with 1+ lab test	84 (3.8%)	1217 (1.7%)
Number of patients with 3+ lab tests	18 (0.81%)	273 (0.38%)
PLATELETS		
Number of lab tests	2646	1,08,722
Number of patients with 1+ lab test	500 (22%)	30,732 (42%)
Number of patients with 3+ lab tests	231 (10%)	11544 (16%)
PROTHROMBIN TIME, P	
Number of lab tests	711	28,007
Number of patients with 1+ lab test	197 (8.8%)	10,446 (14%)
Number of patients with 3+ lab tests	46 (2.1%)	2502 (3.5%)

**Table 9.** Lab test data availability in patients with SARS-CoV-2 PCR testing and longitudinal lab data. Lab test data availability for all patients who underwent SARS-CoV-2 PCR testing in the Mayo Clinic EHR database from February 15, 2020 to May 28, 2020 with longitudinal testing data available (i.e. patient received the same lab test on three separate days within + / − 30 days of PCR testing date). Includes counts of lab tests and counts of patients with 1+ and 3+ lab tests both overall and for selected coagulation-related lab tests (activated partial thromboplastin time, D-dimer, fibrinogen, platelets, and prothrombin time).

	COVID_pos_	COVID_neg_
Total number of patients	246	13,666
Number patents with 1+ test from day −30 to day −1	150 (61%)	11,567 (85%)
Number patents with 1+ test from day 0 to day 30	240 (98%)	13,501 (99%)
Total number of lab tests	89,587	2,634,070
Number of lab tests from day −30 to day −1	8698	763,808
Number of lab tests from day 0 to day 30	80,889	1,870,262
ACTIVATED PTT		
Number of lab tests	355	5186
Number of patients with 1+ lab test	86 (35%)	2722 (20%)
Number of patients with 3+ lab tests	20 (8.1%)	406 (3.0%)
D-DIMER, P		
Number of lab tests	855	1720
Number of patients with 1+ lab test	197 (80%)	1293 (9.5%)
Number of patients with 3+ lab tests	99 (40%)	56 (0.41%)
FIBRINOGEN, P		
Number of lab tests	275	2965
Number of patients with 1+ lab test	81 (33%)	1168 (8.5%)
Number of patients with 3+ lab tests	18 (7.3%)	273 (2%)
PLATELETS		
Number of lab tests	2343	87,517
Number of patients with 1+ lab test	245 (100%)	13,399 (98%)
Number of patients with 3+ lab tests	231 (94%)	11,544 (84%)
PROTHROMBIN TIME, P		
Number of lab tests	676	24,489
Number of patients with 1+ lab test	165 (67%)	7209 (53%)
Number of patients with 3+ lab tests	46 (19%)	2502 (18%)

It is important to note that while we center the study period around the PCR testing date, this date may not correspond to the same disease state of COVID-19 for each individual in the COVID_pos_ cohort. To account for the potential variability in disease progression, we have performed a sensitivity analysis on the time intervals (Table 3). Additionally, there are several covariates that may influence these longitudinal trends and should be explored further. For example, we have already considered whether previous or concomitant administration of anticoagulants or antiplatelet agents influences patient lab test results and/or outcomes. Similarly, in the future, we intend to explore whether longitudinal lab measurement trends differ between outpatient, inpatient, and ICU admitted patient cohorts. New datasets can also be utilized; for example, rather than grouping patients by the identified thromboembolic phenotypes extracted from the clinical notes alone, patients could be stratified by those who had imaging studies (duplex ultrasound, CT scan, etc.) performed, and phenotypes could be directly extracted from these procedural reports. As more data accumulates from COVID_pos_ and COVID_neg_ patients in the coming months, these analyses need to be expanded to assess similarities and differences in the temporal trends of laboratory test results among a wider range of patient subgroups relevant for COVID-19 outcomes, such as those who have pre-existing conditions (e.g. diabetes, hypertension, obesity, malignancies) or patients who are on specific medication (e.g. ACE inhibitors, statins, immunosuppressants).

In summary, this work demonstrates significant progress toward enabling scaled and digitized analyses of longitudinal unstructured and structured EHRs to identify variables (e.g. laboratory results) which are associated with relevant clinical phenotypes (e.g. COVID-19 diagnosis and outcomes). In doing so, we identified trends in lab test results which may be relevant to monitor in COVID-19 patients and warrant both clinical and mechanistic follow-up in more targeted and explicitly controlled prospective analyses.

## Materials and methods

### Study design, setting and patient population

This is a retrospective study of patients who underwent polymerase chain reaction (PCR) testing for suspected SARS-CoV-2 infection at the Mayo Clinic and hospitals affiliated to the Mayo health system. This research was conducted under IRB 20–003278, ‘Study of COVID-19 patient characteristics with augmented curation of Electronic Health Records (EHR) to inform strategic and operational decisions’. For further information regarding the Mayo Clinic Institutional Review Board (IRB) policy, and its institutional commitment, membership requirements, review of research, informed consent, recruitment, vulnerable population protection, biologics, and confidentiality policy, please refer to www.mayo.edu/research/institutional-review-board/overview.

### Longitudinal lab testing tied to COVID-19 PCR diagnostic testing

We analyzed data from 74,586 patients who received PCR tests from the Mayo Clinic between February 15, 2020 to May 28, 2020. Among this population, 2232 patients had at least one positive SARS-CoV-2 PCR test result, and 72,354 patients had all negative PCR test results. In order to align the data for the analysis of aggregate longitudinal trends, we selected a reference date for each patient. For patients in the COVID_pos_ cohort, we used the date of the first positive PCR test result as the reference date (day = 0). For patients with all negative PCR tests, we used the date of the first PCR test result as the reference date (day = 0). We defined the study period for each patient to be 30 days before and after the PCR testing date. Patients with contradictory PCR test results were excluded for the purpose of this analysis; for example, a positive PCR test result and a negative PCR test result on the same day, or a positive PCR test result followed immediately by several negative PCR test results.

Over 4 million test results from 6298 different types of lab tests were recorded for the patients who received PCR tests in the 60-day window surrounding their PCR testing dates at the Mayo Clinic campuses in Minnesota, Arizona, and Florida. Among these lab tests, we restricted our analysis to 194 tests with at least 1000 observations total and at least 10 observations from the COVID_pos_ cohort among the patients with PCR testing on or before May 8, 2020. In addition, we considered different subsets of the COVID_pos_ cohort for the analysis of each of the 194 lab tests, due to differences in availability of testing results. For each lab test, we consider the results from patients with three or more observations during the study period.

In the end, there are 246 SARS-COV-2 positive and 13,666 SARS-CoV-2 negative patients that had three or more test results during the study period for at least one of the assays among the 194 lab tests considered. We take this set of 246 COVID-19 positive patients to be the COVID_pos_ cohort. In order to construct the COVID_neg_ cohort from the 13,666 COVID-19 negative patients, we apply propensity score matching, which is described in the next section.

### Propensity score matching to select the final COVID_neg_ cohort

To construct a COVID_neg_ cohort similar in baseline clinical covariates to the COVID_pos_ cohort, we employ 1:10 propensity score matching (Austin, 2011). In particular, first we trained a regularized logistic regression model to predict the likelihood that each patient will have a positive or negative COVID-19 test result, using the following covariates: demographics (age, gender, race), anticoagulant/antiplatelet medication use (orders for alteplase, antithrombin III, apixaban, argatroban, aspirin, bivalirudin, clopidogrel, dabigatran, dalteparin, enoxaparin, eptifibatide, heparin, rivaroxaban, warfarin in the past year and in the past 30 days), pre-existing coagulopathies (medical history of thrombotic phenotypes including: deep vein thrombosis, pulmonary embolism, myocardial infarction, venous thromboembolism, thrombotic stroke, cerebral venous thrombosis, and disseminated intravascular coagulation from day −365 to day −31 relative to the PCR testing date), and hospitalization status (i.e. whether or not the patient was hospitalized within the past 30 days of PCR testing).

Using the predictions from the logistic regression model as propensity scores, we then matched each of the 246 patients in the COVID_pos_ cohort to 10 patients out of the 13,666 COVID-19 negative patients, using greedy nearest-neighbor matching without replacement (Austin, 2011; Austin, 2014). As a result, we ended up with a final COVID_neg_ cohort that included 2460 patients with similar baseline characteristics to the COVID_pos_ cohort. The characteristics of the two cohorts are summarized in Table 1.

Further, for the analyses conducted on individual lab tests, which include only a subset of patients from the COVID_pos_ cohort, we use the propensity scores to match each patient from the COVID_pos_ cohort to 10 patients from the COVID_neg_ cohort which have the most similar propensity scores and lab tests available. For example, for the fibrinogen lab test, in which we have data on 81 patients from the COVID_pos_ cohort, we select 810 patients from the COVID_neg_ cohort and the most similar propensity scores to be the control group. In this way, we ensure that all of the comparisons are done between subsets of the positive and negative cohorts with similar propensity scores, and therefore similar underlying characteristics.

### Statistical significance assessments for lab test differences over prognostic time intervals for SARS-CoV-2 infection

We conduct a systematic statistical analysis to identify tests that show significant differentiation among the COVID_pos_ cohort during a set of predetermined prognostic time intervals for SARS-CoV-2 infection. In particular, we group the lab test measurements for each patient into the following nine time intervals relative to their date of PCR testing: pre-infection (days −30 to −11), pre-PCR (days −10 to −2), time of clinical presentation (days −1 to 0), and post-PCR phases 1 (days 1 to 3), 2 (days 4 to 6), 3 (days 7 to 9), 4 (days 10 to 12), 5 (days 13 to 15), and 6 (days 16 to 30).

For each lab test and for each of each of our nine pre-specified time intervals, we compared the mean lab test value among patients who underwent at least one such lab test in the COVID_pos_ cohort over that time interval to the mean lab test value in the COVID_neg_ (matched) cohort over that time window. We only considered (lab test, time interval) pairs in which there were at least three patients contributing to laboratory test results in both groups. Specifically, for each (lab test, time interval) pair, we conducted the following procedure:

1. Compute (patient, time interval) averages: We compute the average lab test values for each patient in the COVID_pos_ and COVID_neg_ (matched) cohorts during the specified time interval.
2. Statistical hypothesis testing: We conduct a Mann-Whitney *U* test in order to test the null hypothesis that the average lab test results for each of the (patient, time interval) pairs from the COVID_pos_ and COVID_neg_ (matched) cohorts come from the same distribution. In addition, we compute the Cohen’s D statistic as a measure of the effect size.

Once we have the statistics and p-values for each (test, time window) pair, in order to account for multiple hypotheses, we apply the Benjamini-Hochberg (BH) procedure with FDR controlled at 0.05. The results from the systematic comparisons which met our thresholds for effect size and statistical significance (Cohen’s D > 0.35, BH-adjusted Mann-Whitney p-value <0.05) are shown in Table 2.

### Sensitivity analysis to assess the impact of perturbed clinical time windows

We perform a sensitivity analysis to assess whether or not the key findings from the systematic statistical assessment remain the same if we perturb the considered time intervals. In particular, we repeat the statistical analysis with the time intervals shifted forward or backward 1 day for all patients. For the forward shifted sensitivity analysis, the new time intervals under consideration are: pre-infection (days −30 to −10), pre-PCR (days −9 to −1), time of clinical presentation (days 0 to 1), and post-PCR phases 1 (days 2 to 4), 2 (days 5 to 7), 3 (days 8 to 10), 4 (days 11 to 13), 5 (days 14 to 16), and 6 (days 17 to 30). For the backward shifted sensitivity analysis, the new time intervals under consideration are: pre-infection (days −30 to −12), pre-PCR (days −11 to −3), time of clinical presentation (days −2 to −1), and post-PCR phases 1 (days 0 to 2), 2 (days 3 to 5), 3 (days 6 to 8), 4 (days 9 to 11), 5 (days 12 to 14), and 6 (days 15 to 30). For both the forward and backward sensitivity analyses, we apply the same thresholds of effect size and significance (Cohen’s D > 0.35, BH-adjusted Mann-Whitney p-value <0.05), and we compare the results to the original time intervals.

From this analysis, we observe consistent results (i.e. comparisons meeting same criteria of significance and effect) on (i) both perturbations in 83 out of 130 (64%) lab test trends identified in Table 2 and (ii) at least one perturbation in 114 of 130 (87%) lab test trends. In Table 3, we report the specific results of the time shifted windows for five coagulation-related lab tests (fibrinogen, platelets, prothrombin time, activated partial thromboplastin time, and D-dimer).

### Augmented curation of anticoagulant administration and the coagulopathy outcomes from the unstructured clinical notes and their triangulation to structured EHR databases

A state-of-the-art BERT-based neural network (Devlin et al., 2018) was previously developed to classify sentiment regarding a diagnosis in the EHR (Wagner et al., 2020). Sentences containing phenotypes were classified into the following categories: Yes (confirmed diagnosis), No (ruled out diagnosis), Maybe (possibility of disease), and Other (alternate context, e.g. family history of disease). The neural network used to perform this classification was trained using nearly 250 different phenotypes and 18,500 sentences and achieves 93.6% overall accuracy and over 95% precision and recall for Yes/No sentiment classification (Wagner et al., 2020). Here, this model was used to classify the sentiment around coagulopathies in the unstructured text of the 246 COVID*_pos_* and 13,666 COVID_neg_ patients’ clinical notes, structuring this information so that it could be compiled with longitudinal lab measurement and medication information.

In particular, we used the BERT model to identify the seven coagulopathy phenotypes mentioned in clinical notes in the Mayo Clinic EHR database, including: deep vein thrombosis, pulmonary embolism, myocardial infarction, venous thromboembolism, thrombotic stroke, cerebral venous thrombosis, and disseminated intravascular coagulation. We validated the performance of this model for these phenotypes on a set of 1000 randomly selected sentences from the clinical notes of the patients in the study population. In Table 6, we report the out-of-sample accuracy metrics for the BERT model on this set of sentences, using manually curated labels provided by one of the study’s authors (CP) to be the ground truth. We demonstrate that the model performs well in the task of identifying thrombotic phenotypes in clinical notes, with an overall accuracy of 94.7%, recall of 97.8%, and precision of 92.8%.

## Data Availability

De-identified data will be made available upon reasonable request to the corresponding author (Venky Soundararajan, venky@nference.net).

## Additional files

Transparent reporting form
